# Origin of ω-phase formation in metastable β-type Ti-Mo alloys: cluster structure and stacking fault

**DOI:** 10.1038/s41598-020-65254-z

**Published:** 2020-05-26

**Authors:** Mingjia Li, Xiaohua Min

**Affiliations:** School of Materials Science and Engineering, Dalian University of Technology, Dalian, 116024 P.R. China

**Keywords:** Materials science, Condensed-matter physics

## Abstract

The ω-phase formation and its collapsed structures in metastable β-type Ti-Mo alloys were illustrated by first-principles calculations and experimental evidence of a partially collapsed ω-phase in the nano-scale Mo-depleted region under a rapid cooling via high-angle annular dark-field scanning transmission electron microscopy. The ease of ω-phase formation within -Mo-Ti-Mo- poor cluster structure was not only due to the low energy barrier in the collapse pathway, which was caused by the reduced lattice distortion, but also due to the softening of the shear modulus (*G*_*111*_) as a result of the small charge density difference. The most stable collapsed structure of the ω-phase strongly depended on the minimum stacking fault energy among different collapse degrees in accordance to the smallest charge density difference. Therefore, the concurrent compositional and structural instabilities of the ω-phase was attributed to the coupling effect of the cluster structure with stacking fault from the atomic and electronic basis.

## Introduction

Omega (ω) phase was firstly identified in Beta (β) type Ti-Cr alloys by Frost *et al*. in 1954^1^. Since then, the ω-phase has been observed in numerous group IV transition metals (Ti, Ta, Hf and Zr) and their alloys containing appropriate β-stabilizing elements^2–4^. According to the orientation relationship unanimously described as [0001]_ω_//[111]_β_ and $(11\bar{2}0)$_ω_//$(01\bar{1})$_β_, four crystallographic ω-variants can be found in the β-matrix^5^. The ω-phase can be classified into the deformation-induced ω-phase, athermal ω-phase, and isothermal ω-phase based on its formation process. The morphologies of the ω-phase can be plate-like^6–8^ and ellipsoidal or cuboidal^9–11^, while its sizes range from several to dozens of nanometers depending on both the stressing condition and the compositional partitioning^12–15^. The athermal ω-phase is present in alloys during rapid cooling from the high-temperature single β-phase field^10,11^. Previous works have extensively investigated the effect of ω-phase formation on mechanical and functional properties of metastable β-type titanium alloys^16–21^. As a metastable phase, the ω-phase has attracted the attention of researchers owing not only to its effects on the above properties but also to its complex formation mechanism.

Fontaine *et al*.^22^ reported that the ω-phase can be obtained by submitting the body-centered cubic (bcc) lattice to a 2/3(111) longitudinal displacement wave. They also performed a Monte Carlo simulation to demonstrate that the ω-phase transformation was a product of the linear displacive defects along the [111]_β_ direction. By performing frozen phonon calculations, Ho *et al*.^23^ found that the formation of an ideal ω structure from the β-phase was attributed to the reduction of ground state energy for a 2/3 < 111>_β_ phonon. Lin *et al*.^24^ demonstrated that the athermal ω-phase formation was caused by an atom collapse from the (110)_β_ plane to the (111)_β_ plane along the <111>_β_ direction. Although different mechanisms have been proposed to describe the ω-phase formation, the ω-phase has been widely agreed to form crystallographically via a collapse mechanism that involves various degrees of alternate pairs of {111}_β_ planes to an intermediate position^25^. The collapse degree is evaluated by shear magnitude (*z*), which refers to the atomic moving distance on the (112)_β_ plane along the $[11\bar{1}]$_β_ direction^26–31^. When the collapse is completed, the ω-phase shows a hexagonal structure (*z* = 1/6), whereas a partial collapse leads to a trigonal structure (*z* < 1/6). However, the structural evolution of the ω-phase from trigonal to hexagonal remains under discussion.

When the β-phase stability is low in metastable β-type titanium alloys, the collapse degree is close to 1/6 but is less than 1/6 or even equal to 0 when the stability is relatively or sufficiently high. Hickman^32^ suggested that the ω-phase formed in the solute poor region because of compositional partitioning due to phase separation in Zr-Nb alloys. Liu *et al*.^6^ reported that trigonal and hexagonal ω-phases were simultaneously present due to the inhomogeneity of composition and defect in a water-quenched Ti-9Cr-0.2 O alloy (all contents are expressed as wt.% without special instructions). By coupling aberration-corrected high-angle annular dark-field (HAADF) with the three-dimensional atom tomography (APT) technique, Banerjee *et al*.^10,33^ recently presented direct experimental evidence of phase separation in a rapid-cooling Ti-18Mo alloy and the formation of a partially collapsed ω-phase in Mo-depleted region. Although experimental evidence indicates that the ω-phase formation and its structural evolution strongly depend on alloying element content, the physical nature of the ω-phase formation under poor solute content and its effects on the collapsed structure remain unclear.

Issues related to atomic and electronic levels have been extensively studied in the literature by using first-principles calculations. Lai *et al*.^34^ calculated the energetic pathway of the ω-phase formation in Ti-Nb alloys by shifting the atoms on the {112}_β_ plane along the <111>_β_ direction and found that the energy barrier in the pathway depended on the composition. Niu *et al*.^35^ evaluated the effect of O element on the β to ω transformation in Ti_3_Nb and found that such element increased the energy barrier for the β to ω transformation and the formation energy of the ω-phase. Choudhuri *et al*.^11^ calculated the charge density difference in the supercells of Ti-V alloys and found that ω-phase formation with high V content was suppressed by the strong V-V bond. Banerjee *et al*.^33^ simulated the ω-phase transformation via the collapse mechanism in Ti-Mo alloys and found that a partially collapsed structure was energetically favored over a fully collapsed structure with an Mo content of 8.33 at.%. The abovementioned first-principles calculations were performed by constructing supercells with an uniform or ordered distribution of solute atoms. However, the aforementioned experimental evidence obtained via high resolution electron microscopy and atom probe microanalysis reveals that composition partitioning is ubiquitous in metastable titanium alloys. Alloying elements with cluster structures have been successfully utilized to describe the phase structures in Al-Zr^36^, Au-Pt^37^, and Ti-V^38,39^. In our previous work^40,41^, the -Mo-Ti-Mo- cluster structure was constructed in the supercells of β-phase, α′′-martensite and ω-phase in Ti-Mo alloys. The formation sequence of α′′-martensite and ω-phase is attributed to the competition among several moduli along specific directions, including tetragonal shear elastic constant (*C*′), Young’s modulus (*E*_*100*_), and shear modulus (*G*_*111*_). However, given that only the -Mo-Ti-Mo- cluster structure was considered in our previous work, the ω-phase with a collapsed structure easily formed under a poor solute content is not yet comprehensively understood.

Generally, the collapse mechanism of the ω-phase changes the “ABCABCA” sequence to “ABABA” on the {112}_β_ plane along the <111>_β_ direction^34,42,43^. On the basis of the reported correlation between the ω-phase and {112}_β_ < 111>_β_ twins in Ti-Nb alloys^34,44^, the structural evolution of the ω-phase can be influenced by the stacking fault on the {112}_β_ plane. This issue needs to be solved by focusing on the interaction between cluster structure and stacking fault. The stacking fault energy in face-centered cubic (fcc) alloys has been widely used in evaluating phase stability and deformation mode^45–52^. However, only few studies have examined the stacking fault energy in metastable β-type titanium alloys. Wu *et al*.^43^ used coherent potential approximation to construct a model in Ti-30Nb-3Pd alloy with stacking fault and calculated the energy change during the ω-phase transformation to evaluate the ω-phase structure. By considering the aforementioned nano-scale composition partitioning in metastable titanium alloys and the results of our previous work, the coupling effect of the cluster structure and stacking fault may help reveal the origin of ω-phase formation.

By constructing supercells with cluster structure and stacking fault in metastable Ti-Mo alloys, the stacking fault and formation energies obtained via first-principles calculations were used to evaluate the ω-phase formation and its collapsed structure. Experimental evidence of the nano-scale compositional and structural instabilities of the ω-phase was obtained via aberration-corrected high-resolution scanning transmission electron microscopy in a water-quenched Ti-Mo alloy. The ease of ω-phase formation in the -Mo-Ti-Mo- cluster poor region was mainly due to the softening effect of the shear modulus, whereas its collapse degree could be attributed to the presence of minimum stacking fault energy, which was further discussed in terms of the calculated lattice distortion and change density difference from the atomic and electronic basis.

## Results

**Figure 1:**
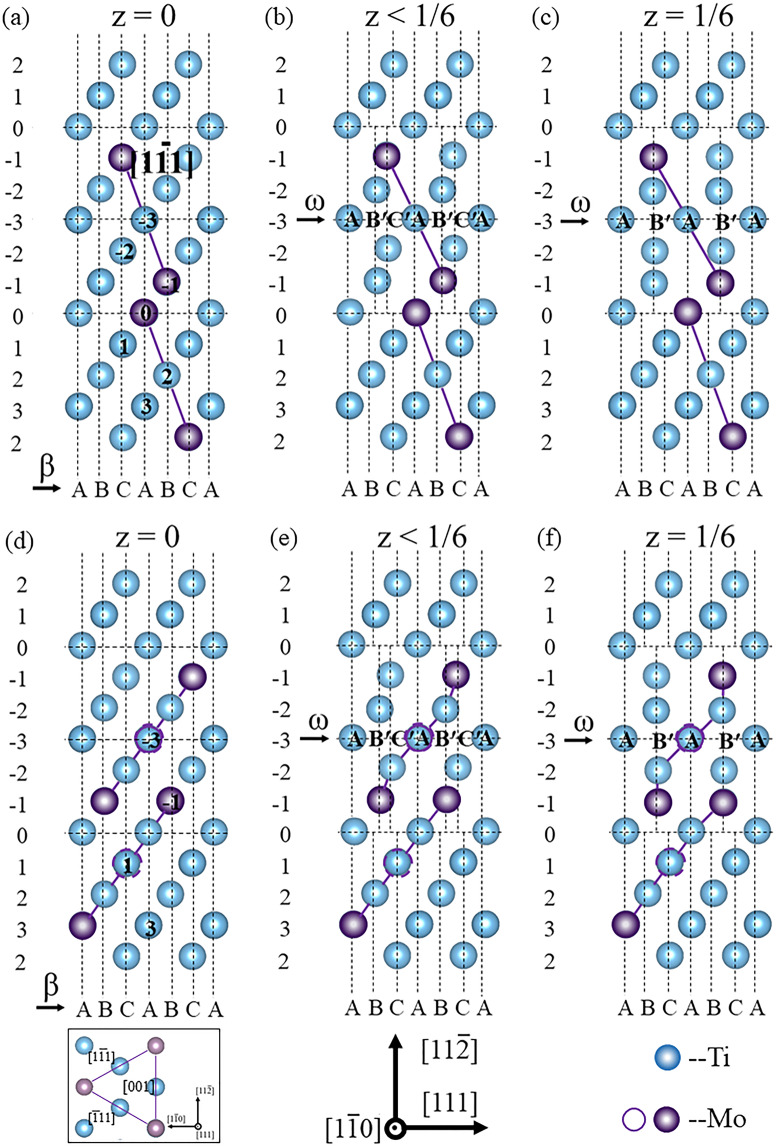
Constructed supercells on ($1\bar{1}0$)_β_ planes with cluster structure and stacking fault. (**a–c**) 2Mo supercell with -Mo-Ti-Mo- linear cluster at two positions (P_−3_ and P_2_) for example; (**d–f**) 3Mo supercell with -Mo-Ti-Mo- trigonal cluster at two positions (P_−3_ and P_1_) for example; (**a,d**) initial β-phase (*z* = 0) with the _(_111)_β_ planes labeled by “ABCA;” (**b,e**) (111)_β_ planes with partial stacking fault (*z* < 1/6) labeled by “AB′C′A;” and (**c,f**) (111)_β_ planes with full stacking fault (*z* = 1/6) labeled by “AB′A”. The blue and purple circles represent the Ti and Mo atoms, respectively. The open and solid circles of the Mo atoms in the 3Mo supercell were located at two adjacent ($1\bar{1}0$)_β_ planes.

Fig. 1(a–f) shows the constructed supercells of the $(1\bar{1}0)$_β_ plane with cluster structure and stacking fault. The {111}_β_ planes in the bcc lattice were denoted by “ABCABCA” (*z* = 0, i.e., β-phase) in Fig. 1(a,d). The adjacent $(11\bar{2})$_β_ planes at positions −1 and −2 (P_−1_ and P_−2_) sheared toward each other, thereby changing “AB′C′AB′C′A” (*z* < 1/6, i.e., partially collapsed or trigonal structure) in Fig. 1(b,e) to “AB′AB′A” (*z* = 1/6, i.e., fully collapsed or hexagonal structure) in Fig. 1(c,f). Fig. 1(a–c) shows the -Mo-Ti-Mo- linear unit along the $[11\bar{1}]$_β_ direction in the 2Mo supercell. The presence of seven positions from P_−3_ to P_3_ indicated that the cluster moved away from the stacking fault. These cluster positions were labeled by the Ti atom in the middle of the linear unit (e.g., the two cluster positions at P_−3_ and P_2_ in the figure). Fig. 1(d–f) shows the trigonal cluster comprising -Mo-Ti-Mo- linear units along the $[1\bar{1}1]$_β_, $[\bar{1}11]$_β,_ and [001]_β_ directions (in side view) in the 3Mo supercell. Four cluster positions from P_−3_ to P_3_, such as the two cluster positions at P_−3_ and P_1_ that were labeled by the Mo atom in the middle of the cluster (the left Mo atom in side view), described the position correlation between cluster structure and stacking fault.

**Figure 2:**
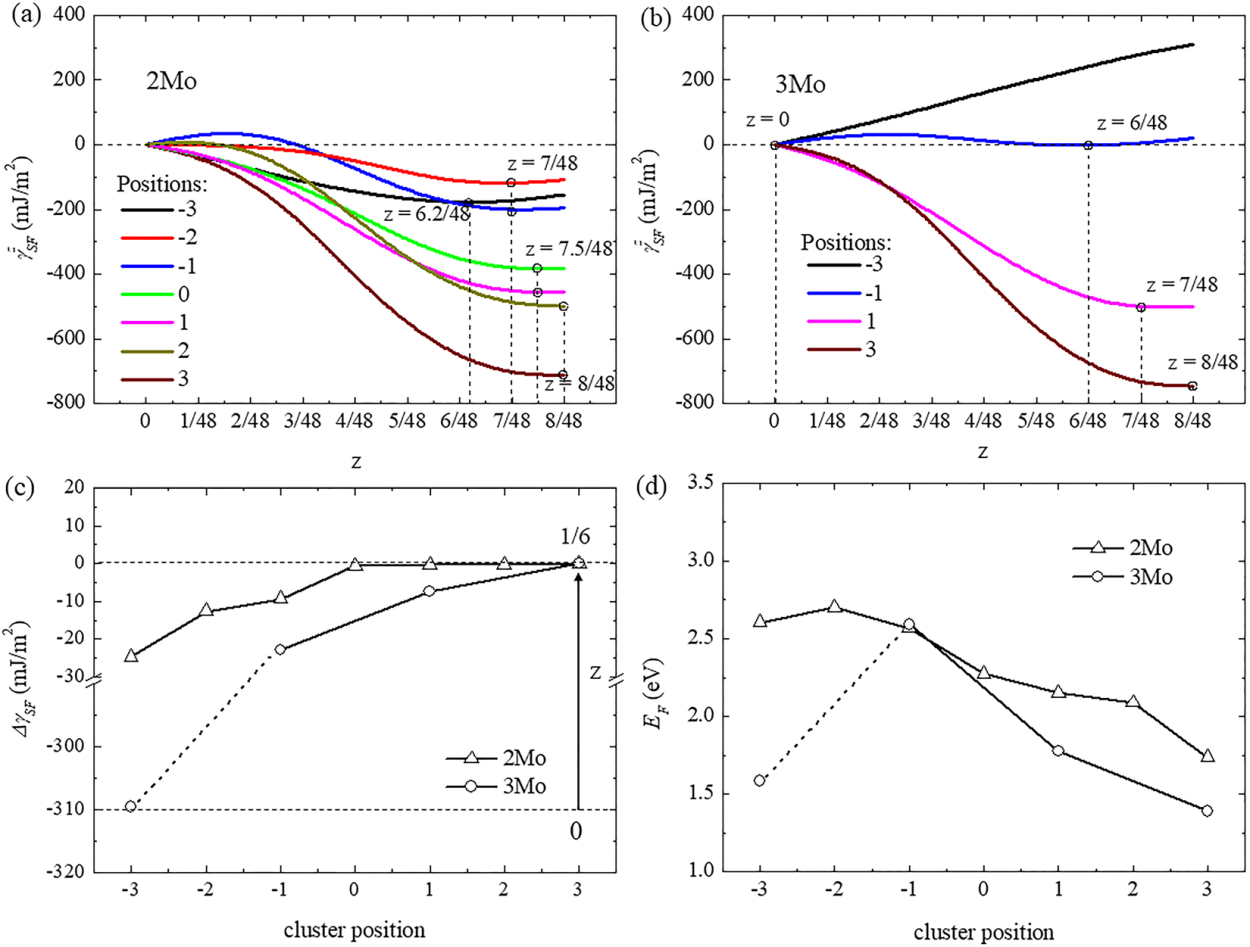
Stacking fault energy (${\gamma }_{{SF}}^{z}$) as a function of collapse degree (*z*) in (**a**) 2Mo supercell and (**b**) 3Mo supercell with different cluster positions. The minimum stacking fault energy corresponding to collapse degree was denoted by the circle in each curve. (**c**) Difference in stacking fault energy (*∆γ*_*SF*_) between a stable structure with minimum stacking fault energy and a fully collapsed structure, and (**d**) formation energy (*E*_*F*_) of a stable structure with minimum stacking fault energy as a function of cluster position in 2Mo and 3Mo supercells.

Fig. 2(a,b) shows stacking fault energy as a function of collapse degree in the constructed 2Mo and 3Mo supercells with different cluster positions. In the initial bcc lattice, no stacking fault energy was observed for all linear and trigonal cluster positions. Fig. 2(a) shows that the stacking fault energy decreases along with collapse degree for each cluster position in the 2Mo supercell. A minimum value (${\gamma }_{{SF}{,}\,{\min }}^{z}$) was observed in each curve as denoted by the circle, thereby suggesting that the corresponding collapsed structure of the ω-phase was the most stable among all collapse degrees. With the linear cluster moving away from the stacking fault from P_−3_ to P_3_, the stacking fault energy decreased, whereas the collapse degree at minimum stacking fault energy increased from 6.2/48 at P_−3_ to 8/48 at P_2_ and P_3_. In the 3Mo supercell with the cluster at P_−3_ as shown in Fig. 2(b), the stacking fault energy increased along with the collapse degree and its minimum value was zero, thereby suggesting that the bcc lattice was the most stable structure without collapse. Similarly, the stacking fault energy decreased along with collapse degree when the trigonal cluster was located far from the stacking fault, and the collapse degree at minimum value increased from 6/48 at P_−1_ to 8/48 at P_3_. To further evaluate the deviation of collapse degree from the trigonal to hexagonal structure of the ω-phase, the difference of stacking fault energy ($\Delta {\gamma }_{{\rm{SF}}}^{z}$) between the stable structure and the fully collapsed structure was obtained as a function of cluster position as shown in Fig. 2(c). The lower $\Delta {\gamma }_{{SF}}^{z}$ indicated that the stable structure of the ω-phase exhibited a smaller collapse degree. In the 2Mo supercell, $\Delta {\gamma }_{{SF}}^{z}$ had the minimum value of −24.7 mJ/m^2^ with a liner cluster at P_−3_, thereby suggesting that the stable trigonal structure had the smallest collapse degree (6.2/48). With the cluster moving away from the stacking fault, the $\Delta {\gamma }_{{SF}}^{z}$ increased to zero at P_2_ and P_3_ corresponding to the stable hexagonal structure. The $\Delta {\gamma }_{{SF}}^{z}$ in the 3Mo supercell exhibited a similarly increasing trend with the trigonal cluster moving away from the stacking fault but was lower than that in the 2Mo supercell at the same position, thereby reducing the collapse degree of the ω-phase, especially at P_−3_ (bcc lattice) with the lowest value of −310 mJ/m^2^. Fig. 2(d) shows the formation energy (*E*_*F*_*)* of the stable structure with minimum stacking fault energy as a function of cluster position in 2Mo and 3Mo supercells. A lower *E*_*F*_ indicated that the cluster structure was more stable in the position of the supercell. The *E*_*F*_ decreased from 2.5 eV to 2.1 eV in the 2Mo supercell from P_−3_ to P_3_ and from 2.5 eV to 1.4 eV in the 3Mo supercell from P_−1_ to P_3_. However, the *E*_*F*_ in the 3Mo supercell at P_−3_ exhibited a low value of 1.6 eV due to the stable bcc lattice. Consequently, the -Mo-Ti-Mo- cluster structures should occupy positions far from the stacking fault, and the stable collapsed structure of the ω-phase changed from trigonal to hexagonal due to the low formation and stacking fault energies.

**Figure 3:**
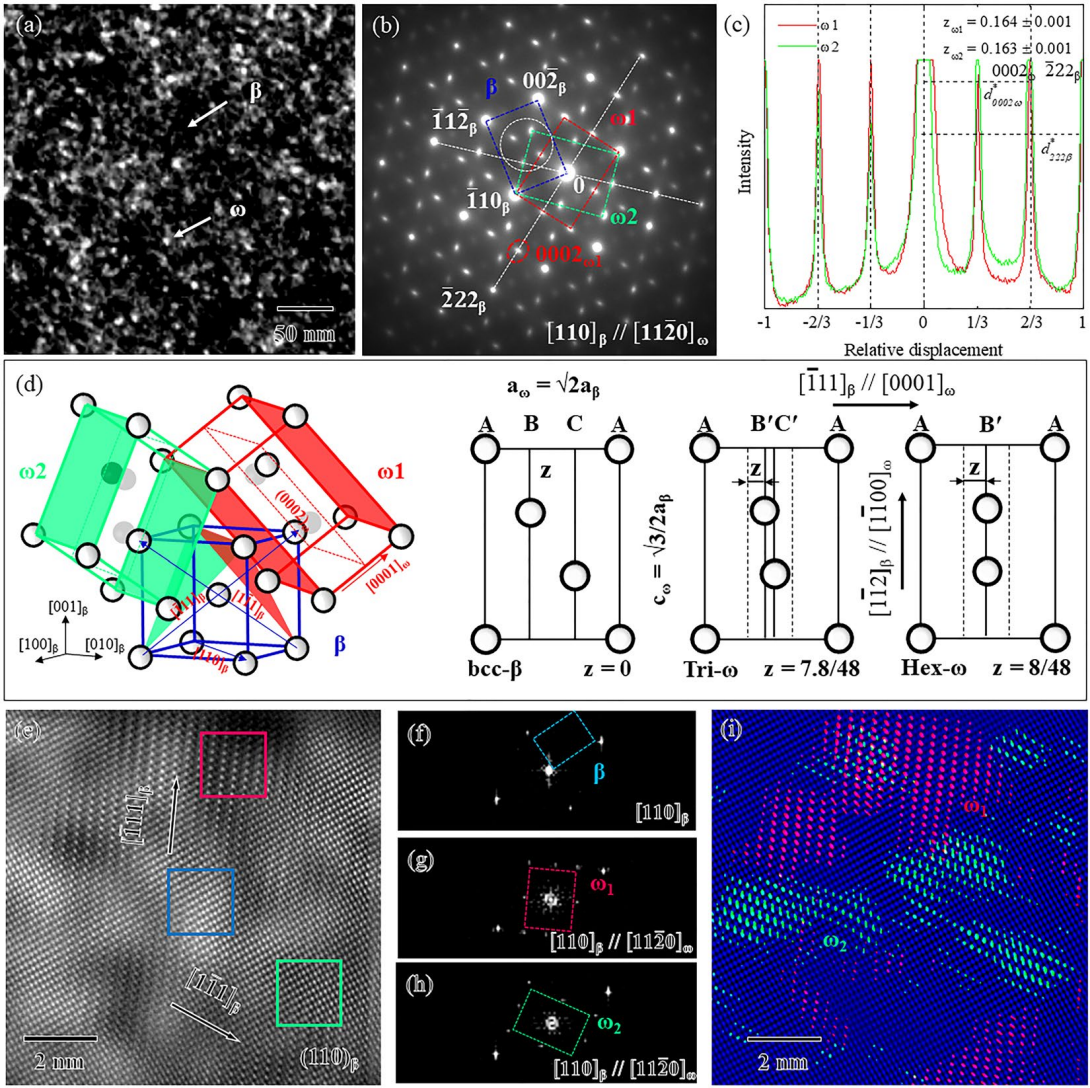
(**a**) TEM dark-field image and (**b**) selected area diffraction pattern of a water-quenched sample in Ti-15Mo alloy; (**c**) intensity profiles of diffraction spots along white lines as shown in (**b**); and (**d**) schematic of the orientation relationships between β-phase and two ω-variations and the structural evolution of the ω-phase. (**e**) Fourier filtered HAADF-STEM; FFT patterns of the (**f**) β-phase, (**g**) ω_1_-variant, (**h**) ω_2_-variant corresponding to regions denoted by the blue, red, and yellow squares in (**e**), respectively, and (**i**) overlay of RGB images filtered by using reflection spots from the ω_1_-variant (R: red), ω_2_-variant (G: green), and β-phase (B: blue). The zone axis is parallel to the [110]_β_ direction.

The TEM dark-field image and its selected area electron diffraction (SAED) pattern were obtained in a water-quenched sample of Ti-15Mo alloy. As shown by the arrows in Fig. 3(a), the black area is mainly β-matrix which may contain a small amount of ω-particles, while the white particles are mainly ω-phase with a diameter of less than 10 nm. The SAED pattern in Fig. 3(b) exhibited three sets of spots along the [110]_β_ zone axis that corresponded to two ω-phase variants of ω_1_ and ω_2_ (denoted by the red and green squares, respectively) as well as the β-phase (denoted by the blue square). The diffraction spots selected to produce the dark-field image was marked by a white circle in Fig. 3(b). The intensity profiles of two ω-variants along the <111>_β_ direction were denoted by the dotted lines and were analyzed on the basis of the SAED pattern in Fig. 3(c). The peak positions of ω-phase spots slightly deviated from relative displacements of 1/3 and 2/3, suggesting the formation of the ω-phase without an ideal hexagonal structure. To quantitatively evaluate its collapse degree, the ratio of ${d}_{{\rm{0002}}{\omega }}^{\ast }$*/*${d}_{{\rm{222}}{\beta }}^{\ast }$ in reciprocal space was obtained by establishing a formula with collapse degree as derived from the Appendix of our previous work^41^. As a result, the ω_1_-variant and ω_2_-variant had collapse degrees of 0.164 ± 0.001 and 0.163 ± 0.001, respectively. In terms of the orientation relationship between the β-phase and ω-phase and the structural evolution of the ω-phase as shown in Fig. 3(d), the ω-phase with a partially collapsed structure was formed in the alloy. The Fourier filtered image in Fig. 3(e) exhibited nano-scale regions with relatively brighter and darker contrasts, which indicated compositional partitioning within the β-matrix. The bright and dark regions corresponded to the Mo-enriched and Mo-depleted regions, respectively. The typical structural features within different regions were further detected by the FFT patterns. Only the β-phase spots in Fig. 3(f) were obtained within the Mo-enriched region (denoted by the blue square in Fig. 3(e)). The two ω-variants (ω_1_ and ω_2_) spots in Fig. 3(g,h) were present in the Mo-depleted regions (denoted by the red and green squares in Fig. 3(e), respectively). The overlay of RGB images was filtered by using reflection spots from the ω_1_-variant (R: red), ω_2_-variant (G: green), and β-phase (B: blue), which corresponded to the Mo-depleted and Mo-enriched regions in Fig. 3(e). The green and red regions indicated that the formed ω-phase had a size of several nanometers. These experimental results indicated that the ω-phase with a trigonal structure was prone to form in the nano-scale Mo-depleted region due to compositional partitioning.

**Figure 4:**
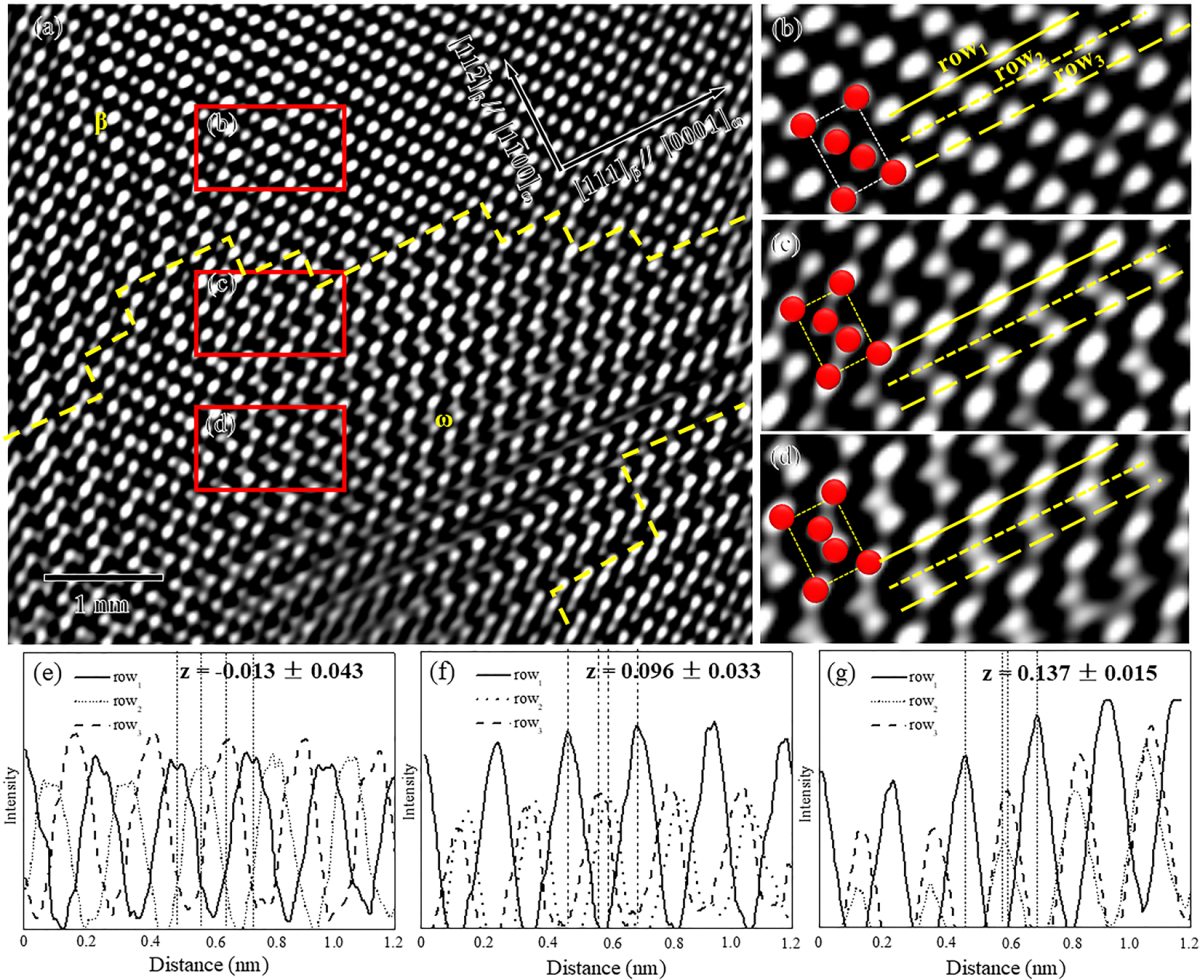
(**a**) Fourier filtered HAADF-STEM image along the <110>_β_ zone axis with the ω-phase and β-matrix distinguished by yellow dotted lines. The enlarged images are indicated by red squares of (**a**) with yellow row_1_, row_2_ and row_3_ along [111]_β_ direction and red motifs. (**b**) in Mo-enriched region, (**c**) near boundary of β-matrix, and (**d**) in Mo-depleted region. (**e–g**) The intensity profiles of the three rows and the collapse degrees (*z*) corresponded to (**b**) to (**d**), respectively.

Fig. 4(a) shows the Fourier filtered HAADF-STEM image of a water-quenched sample of Ti-15Mo alloy along the [110]_β_ zone axis. The ω-phase and β-matrix were distinguished by the yellow dotted lines from the bottom to the top of the image. Three enlarged images (Fig. 4(b–d)) corresponding to the three regions denoted by the red squares in Fig. 4(a) indicated the different collapse degrees of the {111}_β_ plane. The β-matrix in Fig. 4(b) exhibited no collapse in the Mo-enriched region, whereas the ω-phase with partial collapse in Fig. 10(c,d) was close to the boundary of the β-matrix and in the Mo-depleted region. To quantitatively evaluate the collapse degree, a plot of atomic column intensity as a function of distance was obtained along the <111>_β_ direction, and the relative positions of peaks were denoted by the dashed lines. The value of *z* was computed as the ratio of distance that the $(11\bar{2})$_β_ plane moves along the [111]_β_ direction to 1/2 < 111>a_β_. In the Mo-enriched region, the peaks of row_2_ and row_3_ were present at 1/3 and 2/3 positions between two peaks of row_1_ in Fig. 4(e), and the value of *z* was −0.013 ± 0.043. Near the boundary of the β-matrix, the peaks of row_2_ and row_3_ were closed due to the <111>_β_ atomic collapse as shown in Fig. 4(f), and the value of *z* was 0.096 ± 0.033. In the Mo-depleted region, the value of *z* further increased to 0.137 ± 0.015 as shown in Fig. 4(g). Therefore, the collapse degree of the ω-phase continuously increased from the Mo-enriched region to the Mo-depleted region.

## Discussion

The ω-phase that was easily formed in the -Mo-Ti-Mo- cluster poor region and its collapsed structure strongly depended on the present minimum stacking fault energy obtained via theoretic calculations. Moreover, the partially collapsed ω-phase was most likely present in the nano-scale Mo-depleted region according to the results of experimental observations. From the atomic and electronic basis, both lattice distortion and change density difference were calculated to study the reasons behind the ω-phase formation via the cluster structure and its collapsed structural evolution combined with stacking fault. The concurrent compositional and structural instabilities of the ω-phase was also discussed from the view of a metastable “frozen” state of Mo atoms due to the rapid cooling condition.

### ω-phase formation by cluster structure

**Figure 5:**
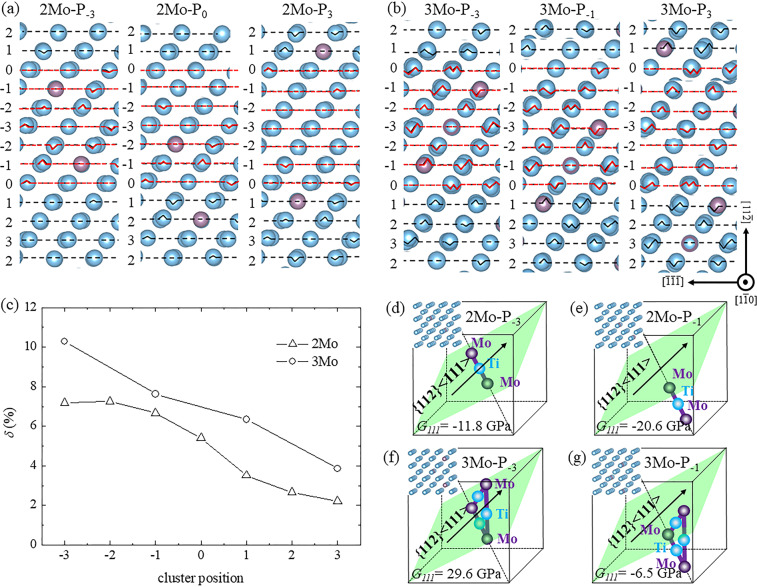
Lattice distortion indicated by up-and-down hills with (**a**) linear cluster positions of P_−3_, P_0_, and P_3_ in the 2Mo supercell; (**b**) trigonal cluster positions of P_−3_, P_−1_, and P_3_ in the 3Mo supercell; and (**c**) change in the lattice distortion (*δ*) of the $[11\bar{2}]$_β_ direction, with the position of cluster structures in the region of supercells denoted by red dotted lines. Schematic of the effect of cluster position on shear modulus (*G*_*111*_) with the constructed 2Mo and 3Mo supercells. (**d**) Linear cluster at P_−3_; (**e**) linear cluster at P_−1_; (**f**) trigonal cluster at P_−3_; and (**g**) trigonal cluster at P_−1_.

On the basis of the aforementioned collapse mechanism of the ω-phase, two factors were identified to influence the collapse process on the {112}_β_ plane along the <111>_β_ direction: one is the extrinsic energy barrier in the pathway, whereas the other is the intrinsic resistance of shear along a specific direction. The energy barrier is affected by the lattice distortion, which suppresses the displacive phase transformation^53^. The lattice distortion in this study was caused by the difference in radius between Mo and Ti atoms, thereby resulting in that the $(11\bar{2})$_β_ plane was not flat with up-and-down hills as shown in Fig. 5(a,b) to supply the energy barriers in the pathway of [111]_β_ direction. In 2Mo and 3Mo supercells, the up-and-down hills in a certain region as denoted by the red dotted lines (i.e., lattice distortion) decreased along with the position of the cluster structure from P_−3_ to P_3_. The trigonal cluster structure in the 3Mo supercell caused a more serious lattice distortion than that of the linear cluster structure in the 2Mo supercell. To quantitatively evaluate the effect of cluster structures, the lattice distortion of the $[11\bar{2}]$_β_ direction (*δ*) was obtained as shown in Fig. 5(c). The value of δ decreased from 7.2% to 2.2% in the 2Mo supercell and from 10.3% to 3.8% in the 3Mo supercell along with the cluster position from P_−3_ to P_3_, respectively. In other words, the extrinsic energy barrier for the collapse process of the ω-phase was reduced within -Mo-Ti-Mo- poor region, thereby promoting the formation of this phase.

Shear resistance is reflected by the shear modulus along a certain direction, and its softening effect can benefit the displacive phase transformation. For example, the softening of tetragonal shear elastic constant (*C*′) with respect to the shear resistance on the {110}_β_ plane along the <110>_β_ direction enhanced the α′′-martensite formation in titanium alloys^41,54,55^. Meanwhile, shear modulus (*G*_*111*_) (i.e., shear resistance on the {112}_β_ plane along the <111>_β_ direction) was associated with the collapse mechanism of the ω-phase. Our previous work^41^ reported that the softening of *G*_*111*_ could benefit the ω-phase formation and strongly depended on the Mo content in metastable Ti-Mo alloys. Therefore, *G*_*111*_ was reduced when the cluster structure moved away from the initial position of P_−3_ in Fig. 1(a,d) and softened much easier with the linear cluster than with the trigonal cluster. To further illustrate the correlation between cluster structure and shear modulus, the supercells containing 54 atoms with -Mo-Ti-Mo- linear and trigonal clusters were used to calculate *G*_*111*_, which can be derived by the elastic constants of the bcc structure^6,41^. Given the size limitations of the constructed supercells, only the two typical cluster positions of P_−3_ and P_−1_ were considered in this study as shown in Fig. 5(d–g). The value of *G*_*111*_ decreased from −11.8 GPa to −20.6 GPa in Fig. 5(d,e) and from 29.6 GPa to −6.5 GPa in Fig. 5(f,g) along with the shifting of cluster position from P_−3_ and P_−1_ in 2Mo and 3Mo supercells, respectively. Therefore, the intrinsic shear resistance for the collapse process was softened within the -Mo-Ti-Mo- cluster poor region.

**Figure 6:**
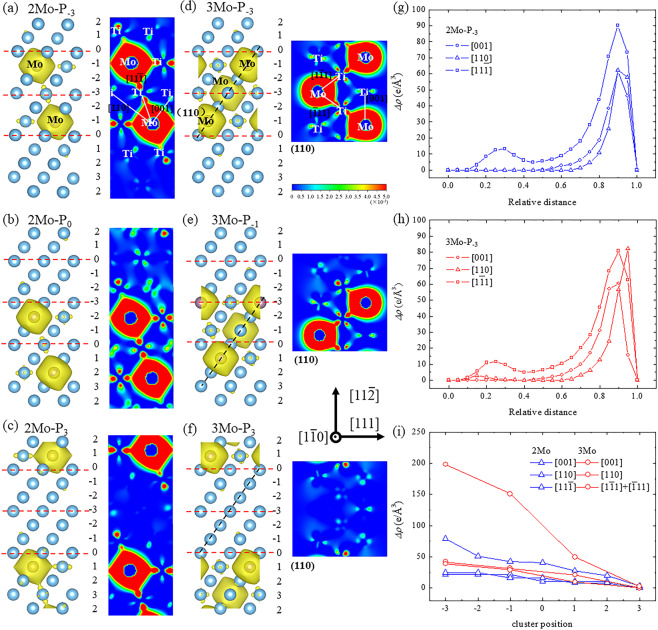
Charge density difference (*∆ρ*) of the {110}_β_ plane in the 2Mo supercell with a linear cluster at (**a**) P_−3_, (**b**) P_0_, and (**c**) P_3_ and in the 3Mo supercell with a trigonal cluster at (**d**) P_−3_, (**e**) P_−1_, and (**f**) P_3_. The line profile of *∆ρ* along the <001>_β_, <011>_β_, and <111>_β_ directions in the (**g**) 2Mo supercell with a linear cluster at P_−3,_ (**h**) 3Mo supercell with a trigonal cluster at P_−3_, and (**i**) integral *∆ρ* in terms of line profile as a function of cluster position.

Shear modulus is also associated with the atomic bonding in adjacent planes, which can be evaluated based on the charge density difference. A larger charge density difference corresponds to a stronger atomic bonding^11^. Gao *et al*.^56^ reported that the charge density difference on the (110) plane along the <111>_β_ direction was high for pure Cr but gradually decreased along with the addition of V, thereby reducing *G*_*111*_. Here, the shear modulus (*G*_*111*_) was influenced by the charge density difference between Ti and Mo atoms along specific directions. Given the -Mo-Ti-Mo- cluster on two adjacent $(11\bar{2})$_β_ planes, the charge density difference (*Δρ*) within the region as denoted by the red dotted lines in Fig. 6 was calculated to evaluate the atomic bonding along the [001]_β_, [110]_β_ and $[11\bar{1}]$_β_ directions, which hindered the shear of $(11\bar{2})$_β_ planes along the [111]_β_ direction. The *Δρ* was indicated with 0.005 e^-^/Å^3^ iso-charge surface in yellow. The *Δρ* in the $(1\bar{1}0)$_β_ plane with a linear cluster (Fig. 6(a)) was large around Mo atoms and exhibited obvious values along the [001]_β_, [110]_β_ and $[11\bar{1}]$_β_ directions, thereby suggesting that a strong Ti-Mo bonding was formed along these directions within the $(1\bar{1}0)$_β_ plane. In the 3Mo supercell, a (110)_β_ plane with a trigonal structure as indicated by the black dotted line in Fig. 6(d) was used to clearly illustrate the charge density difference. In addition, the Ti-Mo bonding was particularly strong in the <111>_β_ direction. To further evaluate the difference of *Δρ* along specific directions, the line profiles of *Δρ* were denoted by the white lines from the Ti atom to the Mo atom in Fig. 6(a,d), and the results are shown in Fig. 6(g,h). The [001]_β_, [110]_β_, $[\bar{1}11]$_β_ and $[1\bar{1}1]$_β_ directions in the 3Mo supercell with a trigonal cluster were analyzed. The *Δρ* along the <111>_β_ direction was the largest, whereas that along the <100>_β_ direction was the smallest, thereby indicating that the Ti-Mo bonding was strongest along the <111> direction, which was in agreement with the findings of Wang^57^. From an electronic basis, this result further revealed the rationality of the -Mo-Ti-Mo- linear and trigonal cluster structures, which was only analyzed based on cohesive energy in our previous work^41^. Similarly, the *Δρ* along specific directions was calculated when the linear cluster in Fig. 6(b,c) and trigonal cluster in Fig. 6(e,f) moved from P_−3_ to P_3_. For the line profiles such as those shown in Fig. 6(g,h), the integral *Δρ* was obtained for each cluster position as shown in Fig. 6(i). The quantitative results indicated that the charge density difference along a specific direction in the 2Mo supercell was lower than that in the 3Mo supercell and decreased as the clusters moved from P_−3_ to P_3_. Therefore, the low charge density difference within the -Mo-Ti-Mo- cluster poor region indicated a reduction in atomic bonding or the softening of the shear modulus, which could benefit the ω-phase formation.

### Collapsed structure of the ω-phase by stacking fault

**Figure 7:**
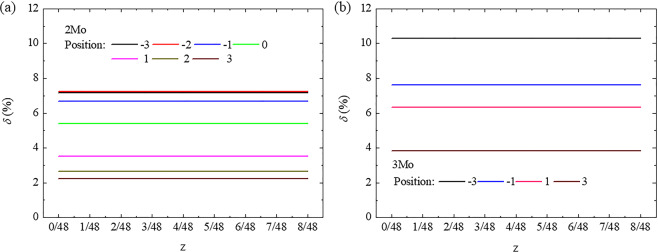
Change of lattice distortion (*δ*) along the $[11\bar{2}]$_β_ direction with collapse degree (*z*) along the [111]_β_ direction on the $(11\bar{2})$_β_ plane in (**a**) the 2Mo supercell with a linear cluster and in (**b**) the 3Mo supercell with a trigonal cluster at different positions.

Our previous theoretic calculations and experimental observations revealed that the ω-phase structure changed from hexagonal to trigonal along with an increasing Mo content^41^. The experimental evidence also indicated that the ω-phase with a trigonal structure was formed in the Mo-depleted region (Fig. 3). Although the aforementioned extrinsic and intrinsic factors were reasonable enough to explain the easy formation of the ω-phase within the -Mo-Ti-Mo- cluster poor region, a direct correlation between the collapse degree of the ω-phase and cluster structure was not clearly revealed. From the collapse mechanism of the ω-phase, the stacking sequence of the {111}_β_ plane in the bcc lattice changes from “ABCABCA” to “ABABA” along the <111>_β_ direction, whereas the structural evolution of the ω-phase can be influenced by the stacking fault on the {112}_β_ plane^34,42,43^. These results also indicated that the stacking fault energy decreased along with the collapse degree and the collapsed structure was the most stable at the present minimum stacking fault energy (Figs. 1 and 2). On the basis of the constructed supercells comprising a cluster structure and stacking fault as shown in Fig. 1(b,c,e,f), both the lattice distortion and change density difference were used to examine the stacking fault energy and collapse degree of the ω-phase. On the one hand, the lattice distortion along the $[11\bar{2}]$_β_ direction was not changed by the shear of atoms along the [111]_β_ direction on the $(11\bar{2})$_β_ plane. For each cluster position in the 2Mo and 3Mo supercells, the value of *δ* did not change along with collapse degree from 0 to 1/6 as shown in Fig. 7, thereby suggesting that the changing tendency of stacking fault energy in Fig. 1(g,h) was not reasonably explained by the lattice distortion.

**Figure 8:**
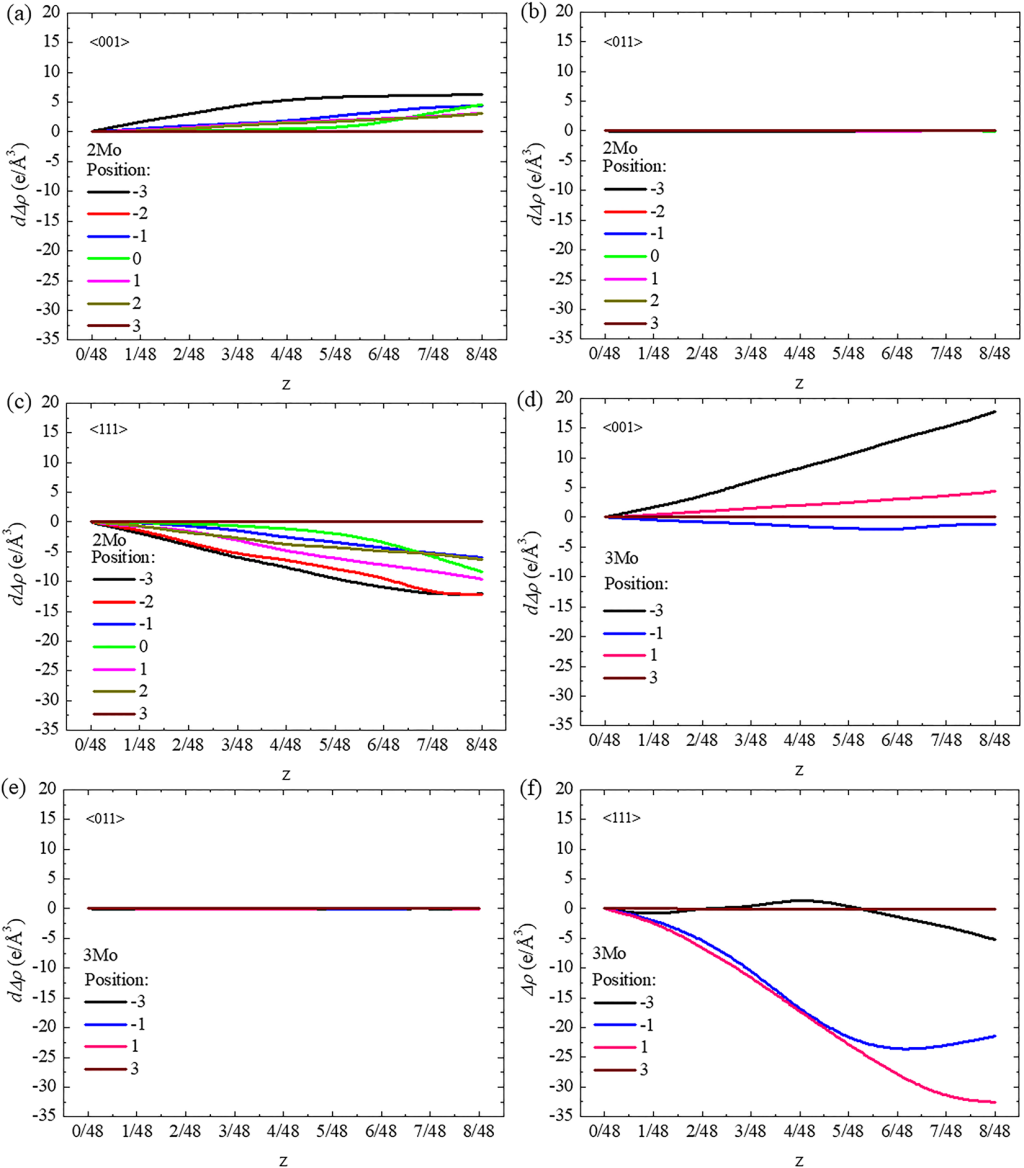
Difference of charge density difference (*d∆ρ*) as a function of collapse degree (z) on the {110}_β_ plane along the (**a**) <001>_β_, (**b**) <011>_β_, and (**c**) <111>_β_ directions in the 2Mo supercell and along the (**d**) <001>_β_, (**e**) <011>_β_, and (**f**) <111>_β_ directions in the 3Mo supercell.

**Figure 9:**
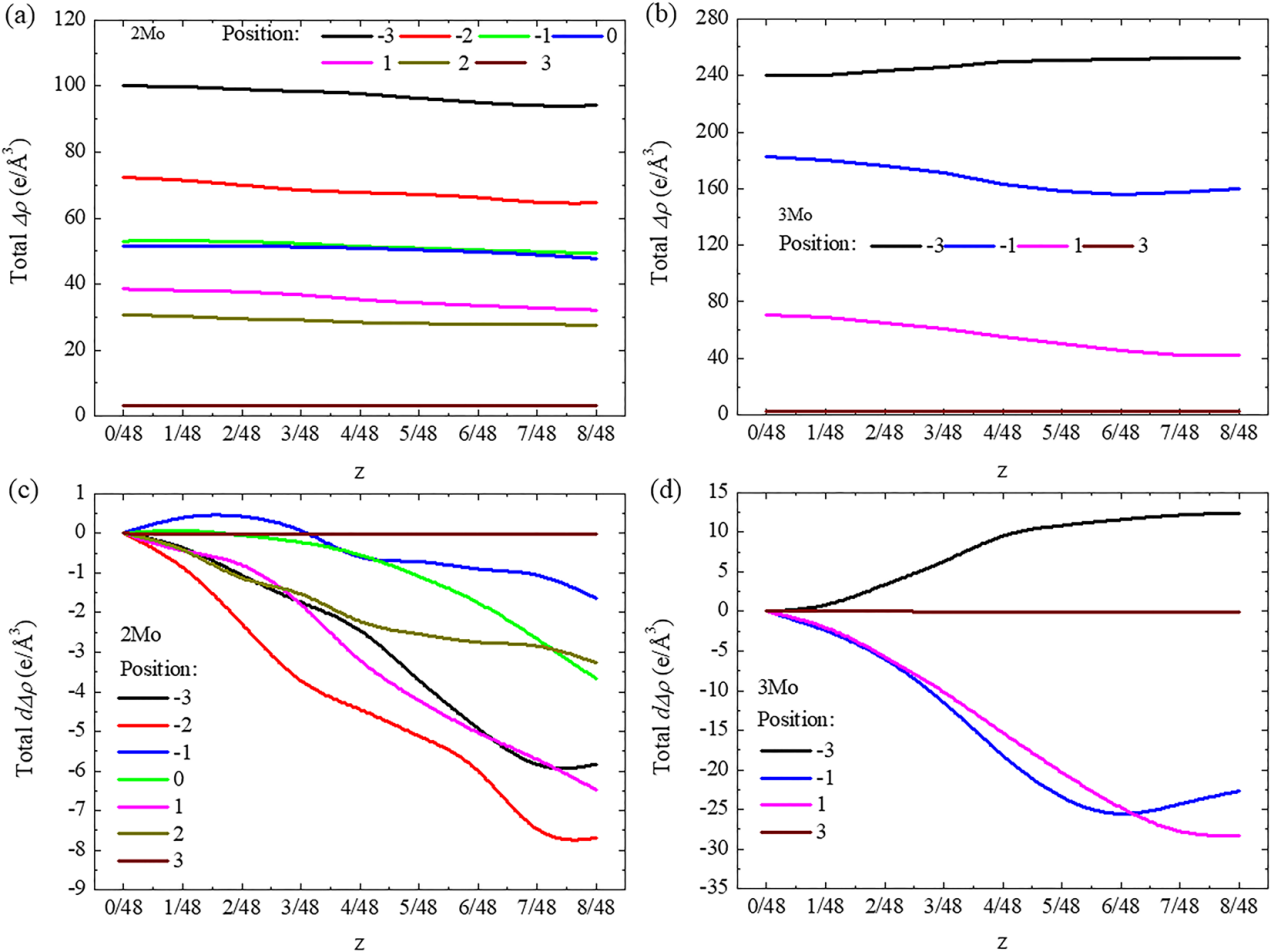
Total charge density difference (total *∆ρ*) in (**a**) the 2Mo supercell and (**b**) the 3Mo supercell, and total difference of charge density difference (total *d∆ρ*) in (**c**) the 2Mo supercell and (**d**) the 3Mo containing <001>_β_, <011>_β_ and <111>_β_ directions on the {110}_β_ plane as a function of collapse degree (*z*).

On the other hand, the stacking fault energy was affected by the charge density difference or atomic bonding, and the variation of stacking fault energy could be analyzed by the redistribution of change density, where some bonding may become either stronger or weaker^58,59^. Shang *et al*.^60^ reported that the larger charge density difference of Al along the $[11\bar{2}0]$ direction led to higher stacking fault energy and shear strength along the $[10\bar{1}0]$ direction in Mg-based alloys. Kioussis *et al*.^61^ found that the variation of stacking fault energy in fcc metals resulted from the change of charge density in three directions across to the {111} slip plane. Compared with the charge density difference without a collapse degree as shown in in Fig. 6, the values of integral *∆ρ* for each cluster position in the 2Mo and 3Mo supercells were calculated at different collapse degrees along specific directions. Here, the difference of *∆ρ* (*d∆ρ*) with and without collapse degree was calculated to describe the redistribution of charge density as shown in Fig. 8. In the 2Mo supercell, the *d∆ρ* along the <100>_β_ direction increased at each cluster position along with an increasing collapse degree but did not show any changes at P_3_ as shown in Fig. 8(a). As shown in Fig. 8(b), the *d∆ρ* along the <110>_β_ direction did not obviously change along with collapse degree at all cluster positions. By contrast, the *d∆ρ* along the <111>_β_ direction exhibited a decreasing trend at each cluster position with an increasing the collapse degree, except at the position of P_3_ as shown in Fig. 8(c). Although the trends of *d∆ρ* (Fig. 8(d–f)) were similar to those recorded in the 2Mo supercell, larger variations in the values of *d∆ρ* were observed along the <100>_β_ and <111>_β_ directions in the 3Mo supercell. To synthetically examine the effect of charge density difference on stacking fault energy, the total values of *∆ρ* and *d∆ρ* along three directions were calculated at different collapse degrees as shown in Fig. 9. The total *∆ρ* decreased for each collapse degree when the cluster structure moved from P_−3_ to P_3_ and decreased along with an increasing collapse degree at each cluster position, except at P_3_ (where no obvious changes were observed) in 2Mo and 3Mo supercells and at P_−3_ (where an increasing trend was reported) in the 3Mo supercell. Moreover, a minimum charge density difference was observed at a collapse degree of less than or equal to 1/6 when the cluster position was near to or far from the stacking fault. The minimum charge density difference corresponded to the zero collapse degree (bcc lattice) in the 3Mo supercell with the cluster at P_-3_. The variations in charge density difference along with cluster position and collapse degree agreed with the trends of stacking fault energy and indicated the collapsed structural evolution of ω-phase, even when the overestimated and underestimated values were considered.

**Figure 10:**
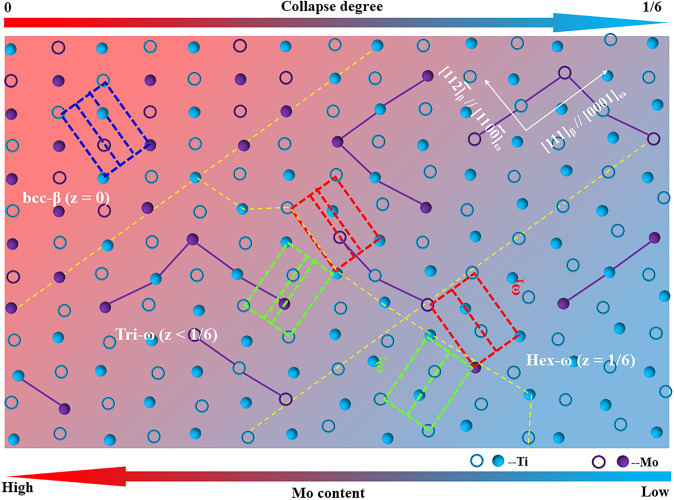
Schematic diagram of the structural evolution of the ω-phase (ω_1_- and ω_2_-variants: red and green motifs) from hexagonal to trigonal in the β-matrix (blue motif) of metastable Ti-Mo alloys with an increasing Mo content and decreasing collapse degree. The open and solid circles were located at two adjacent $(1\bar{1}0)$_β_ planes.

On the basis of the theoretic calculations of stacking fault energy and charge density difference, in the Mo-depleted region, the ω-phase with a hexagonal structure was easily formed, while the experimental evidence confirmed the formation of a trigonal ω-phase due to the compositional instability under the water-quenching condition, that is, a metastable “frozen” state of Mo atoms, thereby leading to the present minimum stacking fault energy (minimum charge density difference) with a partial collapse degree (Figs. 1 and 9). If the Mo atoms sufficiently moved out of the Mo-depleted region, then the collapse degree of the ω-phase would increase up to 1/6, as reported in ref. ^10,33^. However, the formation of an isothermal ω-phase was not the focus of this work. The local instability of the composition is known to be present in quenched β-type titanium alloys, which often leads to local structural instability^10,34,36,62,63^. In addition, the nano-scale compositional partitioning resulted in continuous changes in the solute content between the depleted and enriched regions as shown in Figs. 3 and 4. Therefore, the structural instability of the ω-phase changed continuously due to compositional instability. Consequently, the structural evolution of the ω-phase from hexagonal to trigonal in metastable β-type Ti-Mo alloys was schematically drawn along with Mo content and collapse degree in Fig. 10. The concurrent compositional and structural instabilities of the ω-phase was attributed to the coupling effect of the cluster structure with stacking fault.

In conclusion, the -Mo-Ti-Mo- cluster structures should occupy positions far from the stacking fault in the constructed supercells with a linear cluster along the <111>_β_ direction and a trigonal cluster along the <111>_β_ and <001>_β_ directions. The stable structure of the ω-phase with different collapse degrees on the {112}_β_ plane along the <111>_β_ direction strongly depended on the present minimum stacking fault energy and the formation energy of cluster structures. The ω-phase with a partially collapsed structure was prone to form in the nano-scale Mo-depleted region due to compositional partitioning under a rapid cooling condition. The structural instability of the ω-phase continuously changed along with compositional instability from the Mo-enriched region to the Mo-depleted region due to the metastable “frozen” state of Mo atoms. Within the cluster poor region, the lattice distortion along the <112>_β_ direction on the {112}_β_ plane decreased, thereby leading to the low energy barrier in the collapse pathway. Moreover, the charge density difference corresponding to the atomic bonding along the <111>_β_, <011>_β_ and <001>_β_ directions on the (110)_β_ plane was reduced, thereby softening the shear modulus (*G*_*111*_) along the <111>_β_ direction on the {112}_β_ plane, which benefited the ω-phase formation. The redistribution of charge density exhibited an obvious anisotropy among the <111>_β_, <011>_β_, and <001>_β_ directions with different collapse degrees, and the changing tendency of total charge density difference on the (110)_β_ plane with cluster structure and collapse degree was in accordance with the stacking fault energy and corresponded to the collapsed structural evolution of the ω-phase from trigonal to hexagonal.

## Methods

### Calculation methodology

The calculations were carried out based on density functional theory and by using the Vienna Ab-initio Simulation Package code^64^ and the projector augmented wave method for investigating the core-valence electrons interaction^65^. The exchange correlation potential was measured via generalized gradient approximation and expressed in Perdew-Burke- Ernzerhof form^66^. The plane wave cut-off energy was set at 400 eV. Relaxation was stopped when the energy reached 10^-3^ eV convergence. The Monkhorst-Pack mesh for this system was 9 × 11 × 1. Supercells comprising 24 atoms were constructed with 12 $(11\bar{2})$_β_ planes and 2 and 3 Mo atoms (Ti-15Mo or Ti-22Mo alloys, respectively). According to the collapse mechanism of the ω-phase, the two adjacent $(11\bar{2})$_β_ planes shifted toward each other via *za*_*0*_[111]_β_, where *z* is the collapse degree from 0 to 1/6 with a step of 1/48, and *a*_*0*_ is the lattice parameter of the β-phase. The -Mo-Ti-Mo- linear unit along the $[11\bar{1}$_β_ direction is the most feasible configuration of two Mo atoms^40^ and has seven positions in the supercell. For three Mo atoms, a triangle cluster with three linear units along different directions (i.e., $[1\bar{1}1]$_β_, $[\bar{1}11]$_β_ and [001]_β_) is the most feasible configuration^40^ with four positions in the supercell.

Stacking fault energy (${\gamma }_{{SF}}^{z}$) was calculated as follows in terms of the constructed supercells with cluster structure and stacking fault^48^:

1 $${\gamma }_{{SF}}^{z}=\frac{{E}_{Z}-{E}_{0}}{A},$$

where *E*_*z*_ and *E*_*0*_ are the total energy of a supercell with a collapse degree and the total energy of a perfect bcc supercell, respectively, and *A* is the area of the fault plane that is a cross product of supercell lattice vectors *a*_*0*_$[1\bar{1}0]$_β_ and *a*_*0*_[111]_β_. To further evaluate the stability of an Mo atom occupying a certain position in the supercell, the formation energy (*E*_*F*_) was calculated as

2 $${{E}}_{{F}}={{E}}_{{z}}{-}\,{n}{{E}}_{{Mo}}^{{bcc}}\,{-}\,({24}{-}{n}){{E}}_{{Ti}}^{{hcp}}\,{-}\,{A}{{E}}_{{SF}}^{{Ti}},$$

where ${E}_{{Ti}}^{{hcp}}$ and ${E}_{{Mo}}^{{bcc}}$ are the energies per atom of bulk Ti in the hexagonal close-packed structure and that of Mo in the bcc structure, respectively, *n* is the number of Mo atoms in each supercell, and ${E}_{{SF}}^{{Ti}}\,$is the stacking fault energy without Mo atoms. Following this definition, a lower formation energy corresponds to a more stable Mo atom at a certain position. The formation energy was calculated only in the structure with the minimum stacking fault energy.

The lattice distortion ($\delta$) of each supercell was calculated due to the different radiuses of the Mo and Ti atoms, and the quantitative value of the $(1\bar{1}0)$_β_ plane along the $[11\bar{2}]$_β_ direction was computed as

3 $$D=(x{\prime} -x){\cos }\,{\alpha }_{3}\,{\cos }\,{\alpha }_{1}+(y{\prime} -y){\cos }\,{\alpha }_{2}+(z{\prime} -z){\cos }\,{\alpha }_{3},$$

where *x*′, *y*′, *z*′ and *x*, *y*, *z* are the fractional coordinates of atoms with distortion and non-distortion, respectively, and *α*_*1*_, *α*_*2*_, and *α*_*3*_ are the angles between the [100] and [101], [101] and $11\bar{2}]$, [010] and $[11\bar{2}]$ directions, respectively. The charge density difference (*Δρ* in e^−^/Å^3^) of each supercell was calculated as follows to evaluate the atomic bonding^11^:

4 $$\Delta {\rm{\rho }}={\rho }_{{Ti}{-}{Mo}}-{\rho }_{{Ti}},$$

where *ρ*_*Ti-Mo*_ is the charge density of the supercell containing Ti and Mo atoms, and *ρ*_*Ti*_ is the charge density of the supercell with Mo replaced by Ti atoms. The charge density difference on the $(1\bar{1}0)$_β_ plane along the <001>_β_, <011>_β_ and <111>_β_ directions was quantitatively evaluated.

### Material preparation and microstructural observation

Ti-15Mo alloy was prepared via cold crucible levitation melting. The ingot weighed approximately 1 kg and was homogenized at 1273 K for 3.6 ks, hot forged into a block at 1273 K, hot rolled into a plate with dimensions of 290 mm (*l*) × 50 mm (*w*) × 10 mm (*t*) at 1173 K, and eventually air cooled. After cutting into 10 mm (*l*) × 10 mm (*w*) × 5 mm (*t*), the plate was solution treated for 3.6 ks at 1173 K and underwent water quenching. Our previous works^67,68^ reported that the chemical compositions of Ti-Mo alloys were controlled well by using this melting method and that each alloy had an oxygen content of approximately 0.1 wt.% with small amounts of nitrogen and carbon.

The sample of 10 mm (*l*) × 10 mm (*w*) × 1 mm (*t*) was cut from the solution-treated plate via wire cut electrical discharge machining and then mechanically ground to 0.1 μm in thickness. Subsequently, the foils for microstructural characterization were thinned via dimple grinding (Gatan 656 Dimple Grinder) and ion milling (Gatan PIPS II 695). TEM and atomic resolution HAADF-STEM observations were carried out by using a 300 kV probe corrected electron microscope (FEI Titan G2). HAADF images were captured by using a probe with a convergence semi-angle of 21.4 mrad by setting the camera length at 100 mm. The inner and outer detection angles were 76 mrad and 200 mrad, respectively. To illustrate the structural features at the atomic level, Fourier filtering processing was performed by using a digital micrograph software to remove noisy background from raw images, and the local structural information was analyzed by Fast Fourier Transform (FFT) patterns.
